# Longitudinal Investigation of Psychotherapy Outcomes (LIPO): Description of the Study Protocol

**DOI:** 10.3389/fpsyt.2019.00212

**Published:** 2019-04-08

**Authors:** Leonardo Gonçalves, Guillherme Kirsten Barbisan, Cinthia Danielle Araújo Vasconcelos Rebouças, Neusa Sica da Rocha

**Affiliations:** Post-Graduation Program in Psychiatry and Behavioral Sciences, Universidade Federal do Rio Grande do Sul, Hospital de Clínicas de Porto Alegre, Porto Alegre, Brazil

**Keywords:** psychotherapy, longitudinal studies, interpersonal psychotherapy, psychoanalytical psychotherapy, cognitive–behavioral therapy

## Abstract

**Background:** Despite extensive research in the field of psychotherapies, few studies have compared the primary psychotherapies of naturalistic design, which represents real-life situations.

**Objective:** The objectives of this study were to evaluate three modalities of evidence-based psychotherapy for clinical, psychosocial, and biological outcomes and to identify the mediators and confounders of this process. Our primary hypothesis is that all psychotherapies will improve clinical and psychosocial outcomes and will increase BDNF levels.

**Methods:** *Design:* longitudinal, naturalistic. *Participants:* One hundred twenty-six patients who underwent one of three evidence-based modalities of individual psychotherapy [psychodynamic psychotherapy (PDT), interpersonal psychotherapy (IPT), and cognitive–behavioral psychotherapy (CBT)] were included. *Measure:* Primary outcomes are divided into three domains of variables: clinical (general psychiatric symptoms), biological (serum BDNF levels), and psychosocial (resilience, quality of life, coping strategies, social support, and quality of life-adjusted years of life). Confounding/mediator variables included clinical (personality traits, type of psychotherapy, number of sessions, concomitant use of pharmacological treatment, history of previous psychotherapeutic treatment, medical and psychiatric comorbidities, and psychiatric diagnosis), psychosocial (psychosocial stressors, therapeutic alliance, and defense mechanism style), and other (religiosity) factors. *Procedure:* The follow-up period will be baseline and 6 months and 1 year after entering the study. The study will include 42 controls for biological variables only. Sample size calculation considered a significance level of 5% and a power of 80% to detect a difference of 0.22 with a standard deviation of 0.43, assuming losses of 20–30% of patients. The comparison between the modalities of psychotherapy will be by generalized estimating equations (GEE) model, the analysis of mediators by the Hayes method, and confounders by multivariate logistic regression.

**Discussion:** The findings of this study are intended to demonstrate the outcomes of evidence-based psychotherapies for clinical, psychosocial, and biological parameters and to understand the mediators and confounders of this process in a real-life setting for patients with severe mental illness, thus contributing to the establishment of evidence-based public health policies in the field of psychological interventions.

## Introduction

Psychotherapies are effective interventions for most mental disorders (1–4). A systematic review of 61 meta-analyses (852 clinical trials comprising a total of 137,126 participants) examined the effect of pharmacotherapy and psychotherapy for major psychiatric disorders. This review revealed that the effect sizes of psychotherapies tended to be larger [0.58 (95% CI, 0.40–0.76)] than those of pharmacotherapy [0.40 (95% CI, 0.28–0.52)]. This investigation concluded that more support from public funding agencies is necessary for studies of psychotherapy (4).

Comparisons between different types of psychotherapy have long been a source of controversy (5), even with subsequent reproductions of meta-analyses (6, 7). This situation persists because of the dodo bird effect, i.e., that different techniques present similar effect size outcomes. The issue becomes more complex given that most studies do not have sufficient power to detect differences between psychotherapies. Furthermore, meta-analyses are composed of clinical trials with a considerable risk of bias (8).

Although there have been many randomized clinical trials (RCTs) evaluating the outcomes of psychotherapies, this is the first protocol of a study to evaluate main available evidence-based psychotherapies using a naturalistic longitudinal design and including patients referred from primary and secondary centers with complex and severe disorders. This design has the advantage of evaluating clinical outcomes in a real-world situation. Clinical trials, on the other hand, have the standardization of interventions, but can suffer from a lack of rigorous control related to the criteria for inclusion of patients; also, longitudinal studies have a level of evidence lower than that of randomized clinical trials and meta-analyses (9). To our knowledge, this is the first study with this design, because a Swedish naturalistic study by Werbert et al. compared three psychotherapies but used public health system records. Of the 1,498 patients, only 180 remained in the study, and the records were obtained mostly online. The study showed that there was no significant effect of the type of therapy, duration, or effect of the therapist, despite the limitations.

Reviewing the longitudinal studies in psychoanalytical psychotherapy published on PubMed, PsychInfo, and Embase databases within the last 5 years, we found seven studies (10–16), all of which were European. These studies were conducted mostly on patients with personality disorder, anxiety disorders, and depressive disorders. To the best of our knowledge, no published studies have followed up patients who underwent different kinds of psychotherapy.

The question of how to evaluate outcomes in psychotherapy is quite complicated and has been the subject of numerous studies (17). The most commonly used outcome measures in recent studies have included general psychiatric symptoms (10, 14), anxiety and depressive symptoms (13), coping and defense mechanisms (12), quality of life (16), and quality of life-adjusted years of life [Short-Form Six-Dimension (SF-6D)] (10).

Psychotherapies can function by environmental epigenetic mechanisms, altering gene expression through methylation of the DNA of the serotonin transporter gene (5HTT) and altering imaging exams. However, this subject has been rarely studied (18). The search for biological markers of mental disorders has advanced in recent years, and this work has revealed neurotrophins. These neurotrophins, particularly brain-derived neurotrophic factor (BDNF), appear to be involved in the pathophysiological basis of many neurodegenerative and psychiatric disorders (19). BDNF is a neurotrophin distributed largely by the central nervous system that is involved in neuron growth, development, and plasticity (20, 21). It is associated with both mental disorders and physiological states such as sleep and alterations after interventions like electroconvulsive therapy (ECT) and antidepressants (19, 22).

Changes in BDNF have already been investigated in different psychotherapies (23) in clinical (24–26) and experimental contexts (27, 28), with contradictory results.

Patients with borderline personality disorder who underwent behavioral dialectical psychotherapy had a change of methylation of the BDNF gene at the end of the treatment that was associated with change scores in depression, hopelessness, and impulsivity (23). Patients with panic disorder who underwent cognitive–behavioral psychotherapy (CBT) and had a poor response had significantly lower serum BDNF than patients with a good response (29).

Despite the extensive literature of comparative studies of psychotherapies, there are still issues related to the effects of moderators and mediators in the psychotherapeutic process, especially considering a view of protective factors, biological factors, and outcomes that encompass factors of positive psychiatry such as quality of life and resilience. In addition, further studies are needed in naturalistic and severe patient contexts that assess psychotherapy in practice, seeking external validity.

## Aims

Our primary aim is to evaluate the outcomes associated with different forms of individual psychotherapy [psychodynamic psychotherapy (PDT), interpersonal psychotherapy (IPT), and CBT] in a public outpatient clinic for mental disorders. Our primary outcomes are divided into three domains of variables: clinical (general psychiatric symptoms), biological (serum BDNF levels), and psychosocial (resilience, quality of life, social support, and quality of life-adjusted years of life).

Our secondary aim is to evaluate potential confounders/mediators/moderators for the main outcomes: personality traits, type of psychotherapy, number of sessions, the therapist’s duration of training, concomitant use of pharmacological treatment, history of previous psychotherapeutic treatment, medical and psychiatric comorbidities, psychiatric diagnosis, therapeutic alliance, and defense mechanisms.

Our main hypothesis is that all three modalities of therapy will produce reduction of symptoms in the clinical domain and improvement in the psychosocial domain. All psychotherapies will increase BDNF at 6 months of follow-up. As secondary hypotheses, the mechanisms of improvement will be mediated by the therapeutic alliance, motivational status, and defense mechanisms (for PDT). The confounders of this therapeutic improvement will be number of sessions, comorbidities, and medication use. The moderating factors will be gender, age, personality traits, religiosity, and defense mechanisms (for CBT).

## Methods

### Design

The Longitudinal Investigation of Psychotherapy Outcome (LIPO) study will compare three modalities of psychotherapy (PDT, CBT, and IPT) in a longitudinal course, in a naturalistic setting. The choice of the three modalities of psychotherapy is evidence-based and listed in guidelines for indication of first-line treatment for depression (30, 31).

The evaluations will take place in three moments: in the baseline (until the fourth session of psychotherapy) and 6 and 12 months after entering the study. The invited participants will be the patients who are undergoing any psychotherapy in the outpatient clinic of the Hospital de Clínicas of Porto Alegre (HCPA).

### Participants

All patients included at the HCPA psychotherapy clinic will be evaluated by a fourth-year psychiatry resident with training in psychotherapy. Patients will be referred from the outpatient clinic of clinical psychiatry, pain medicine, or preoperative bariatric surgery. Each patient who agrees will undergo up to five evaluation interviews and engage in discussions with a supervisor. They will be next referred, based on clinical judgment, to the most suitable modality of psychotherapy. The clinic offers three methods of individual psychotherapy: PDP, IPT, and CBT.

The psychotherapy modality will be indicated by general criteria (17), considering the following characteristics: previous favorable experience with some kind of psychotherapy, psychological-mindedness, presence of personality disorder or traits, number of diagnoses of psychiatric disorders, and relief of symptoms. When diagnosed with personality disorder associated with one or more major psychiatric disorders and psychological-mindedness, PDT will be indicated. When there are up to two major psychiatric diagnoses without personality disorder, it will be directed to CBT as well as in the exclusive quest for symptom relief. When the focus of the problem is exclusively up to two of the following: 1) dispute, 2) roles dispute, 3) role transition, or 4) interpersonal deficits and no personality disorder, IPT will be indicated.

Psychotherapeutic care will be provided by psychiatry residents in their second, third, and fourth years of residency in psychiatry under weekly supervision of dialogued interviews. The residents have weekly theoretical seminars related to each of the psychotherapeutic techniques offered throughout the period of residence. They also engage in weekly clinical seminars in which patients are interviewed by teachers to prompt discussions about the clinical aspects related to cases. When there is doubt about patient management, the patient is interviewed by the supervisor.

### Measures

#### Outcomes and Instruments

For the purposes of this study, outcomes were divided into clinical, psychosocial, and biological domains to make the study as comprehensive as possible in understanding the complex processes of change that may occur in psychotherapies (Table 1). The clinical domain refers to the more traditional measures of symptoms used to mediate the effectiveness of psychotherapy (13, 14). The psychosocial domain relates to the most current tendency of understanding the impact of an intervention on a patient’s functionality, general well-being, and quality of life (10). The biological domain will evaluate the impact of psychotherapy on BDNF (23, 32).

**Table 1:** Assessments according to time of psychotherapy.

Outcomes/confusion factors	Baseline	6 months	12 months
1. Primary outcomes			
1.1 Clinical domain			
Depression symptoms (BDI)	X	X	X
Anxiety symptoms (BAI)	X	X	X
Psychiatric symptoms (SCL-90-R)	X	X	X
1.2 Biological domain			
Serum BDNF	X	X	
1.3 Psychosocial domain			
Quality of life (WHOQOL-BREF)	X	X	X
Resilience (CDRISC)	X	X	X
Social support (MOS)	X	X	X
Quality of life-adjusted years (SF-6D)	X	X	X
2. Confusion/mediator factors			
2.1 Clinical domain			
Diagnosis (review of medical records)	X		
Psychodynamic Diagnosis (OPD-II)	X	X	
			
Comorbidities	X		
Pharmacological treatment	X	X	X
Personality traits (PID-V)	X		X
Number of sessions		X	X
Type of psychotherapy	X		
2.2 Psychosocial domain			
Psychosocial stressors (LEQ)	X	X	X
Therapeutic alliance (CALPAS)	X	X	X
Motivational status (URICA)	X	X	X
Defense mechanism style (DSQ-40)	X	X	X
			
2.3 Others			
Religiosity (DUREL/WHOQOL-SRPB BREF)	X	X	X

BDI, Beck Depression Inventory; BAI, Beck Anxiety Inventory; SCL-90-R, Symptom Checklist-90—Revised; BDNF, brain-derived neurotrophic factor; WHOQOL-BREF, World Health Organization Quality of Life BREF; CD-RISC-25, Connor–Davidson-25 Scale of Resilience; MOS, the Medical Outcomes Study; SF-6D, Short-Form Six-Dimension; OPD-II, Operationalized Psychodynamic Diagnosis; PID-V, the Personality Inventory for DSM-5; LEQ, the Life Events Questionnaire; CALPAS, California Psychotherapy Alliance Scale; URICA, The University of Rhode Island Change Assessment; DSQ-40, The Defense Style Questionnaire; DUREL, Duke University Religion Index; WHOQOL-SRPB-BREF, World Health Organization Quality of Life BREF—Spirituality.

##### Domain of Clinical Variables

###### Psychiatry Symptomatology—General Symptomatology

The Symptom Checklist-90-Revised (SCL-90-R) is a self-administered scale consisting of 90 items distributed over nine dimensions: somatization, depression, interpersonal, obsessive–compulsive sensitivity, anxiety, hostility, phobic anxiety, paranoid ideation, and psychoticism. The scales of answers range from 0 (not at all) to 4 (an extreme degree of injury). Over the 6 months after discharge from the clinical follow-up, only paranoid ideation and the psychosis domains are considered. This situation results in a total of 16 items. The next follow-ups are conducted face to face, which allows for the application of the entire checklist (33).

###### Symptoms of Anxiety and Depression: Beck Depression Inventory and Beck Anxiety Inventory (BAI)

These self-report rating inventories, developed by Beck, aim to measure symptoms of depression and suicidal ideation [Beck Depression Inventory (BDI)] and anxiety [Beck Anxiety Inventory (BAI)] (34).

##### Domain of Biological Variables

###### Serum Levels of BDNF

*Blood Collection* Serum samples will be obtained by venipuncture, and the blood will be immediately centrifuged at 3,000 × *g* for 5 min. The samples will be stored at −80°C until biochemical analysis.
  The levels of neurotrophins (BDNF) will be measured using a sandwich ELISA kit (enzyme-linked immunosorbent assay; Elabscience^®^, Houston, TX, USA), according to the manufacturer’s instructions, with techniques standardized by our group. The total quantity of proteins was measured according to the Lowry method.
*Storage and Analysis of Blood Samples* The laboratory tests will be conducted at the Molecular Psychiatry Laboratory. The samples were stored in an appropriate laboratory managed by Gupo de Pesquisa e Pós-Graduação do Hospital de Clínicas de Porto Alegre (GPPG–HCPA).

##### Domain of Psychosocial Variables

###### Quality of Life

The World Health Organization Quality of Life—BREF (WHOQOL-BREF) is an abbreviated quality of life instrument developed by the World Health Organization. It consists of 26 items that are answered in response scales ranging from 1 to 5, and it is based on a four-domain structure (physical, psychological, social, and environmental) (35, 36).

###### Resilience

The Connor–Davidson Resilience Scale (CD-RISC) is a widely used self-report consisting of a 25-item questionnaire for evaluating individual resilience. It is divided into four domains: tenacity, adaptability–tolerance, reliance on support from the outside, and intuition (37, 38).

###### Social Support

The Medical Outcomes Study (MOS) Social Support Survey consists of 20 self-reported items (39).

###### Quality of Life-Adjusted Years

The SF-6D was adapted to Brazilian Portuguese–Brazil (version 2002). This self-administered instrument produces a unique score, which ranges from 0 to 1. The score represents the strength of an individual’s preference for a given health condition; 0 corresponds to the worst health status and 1 corresponds to the best health status (40).

#### Confusion/Mediator/Moderator Factors

The mediating factors will be therapeutic alliance, motivation, and defense mechanisms (for PDT only). The moderating factors will be gender, age, religiosity, stressors, social support, personality traits, and defense mechanisms (for CBT and IPT). The confounding factors considered will be diagnosis, medication use, number of sessions, and comorbidities.

##### Domain of Clinical Variables

###### Diagnosis

The psychodynamic diagnosis was based on Operationalized Psychodynamic Diagnosis (OPD II), a multiaxial diagnostic system for psychodynamicaly oriented therapists and psychiatrists (41, 42).

###### Personality Assessment

The Personality Inventory for DSM-5 (Diagnostic and Statistical Manual of Mental Disorders) (PID-5)—Adult (43). A set of 25 core elements of personality description that combine in five broad domains of maladaptive personality variation: negative affect, detachment, antagonism, disinhibition, and psychoticism (43).

##### Domain of Psychosocial Variables

###### Psychosocial Stressors

The Life Events Questionnaire (LEQ) evaluates the occurrence of 14 stressful life events over the last 12 months and the impact of these events on the subject’s life. The LEQ also focuses on the stressors’ association with the onset of current psychiatric problems (44).

###### Therapeutic Alliance

The California Psychotherapy Alliance Scale (CALPAS) is a 24-item self-applicable questionnaire (45).

###### Defense Mechanism Style

The Defense Style Questionnaire (DSQ-40) is a 40-item self-applicable questionnaire that aims to identify derivatives of defense mechanisms (46).

###### Motivational Status

The University of Rhode Island Change Assessment (URICA) assesses motivation for change by providing scores on four stages of change: precontemplation, contemplation, action, and maintenance (47).

##### Domain of Other Variables

###### Religiosity

The Duke University Religion Index (DUREL) is a five-item measure of religious involvement. It was developed for use in large cross-sectional and longitudinal observational studies (48, 49). The World Health Organization Quality of Life Group—Spirituality, Religion, and Personal Beliefs (WHOQOL-SRPB BREF) is an abbreviated version of the WHOQOL-SRPB that assesses spiritual, religious, and personal beliefs within quality of life; respondents choose the most suitable item from each of the eight SRPB facets and the one spirituality item (*meaning of life*) located in the psychological domain of the WHOQOL-BREF (36, 50).

#### Sample Size Estimation

Our sample size calculation relied on the study of Schaf (51) using BDNF serum levels before and after psychotherapy as outcomes. We arrived at a number of 42 subjects for each psychotherapy and control group, considering a significance level of 5% and a power of 80% difference to be detected 0.22 with a standard deviation of 0.43 and losses of 20–30%. We estimated a total sample size of 126 patients and 42 blood donor healthy controls for biological variable (as comparative of BDNF samples). The inclusion criteria will include having started psychotherapy within 1 month, an age above 18 years, and a BDI score greater than 15. The exclusion criteria will include psychotic disorders, current use of psychoactive substances, cognitive deficits, and dementias.

The study will include a subsample of healthy controls for control of blood samples that were recruited from the HCPA blood bank. The exclusion criteria for the control group included conditions (current or past) that precluded participation in the study (e.g., psychiatric disorders and/or psychiatric treatments), immune disorders, use of immunosuppressants, anti-inflammatory drugs used for fewer than 3 weeks, smoking, abuse and/or drug addiction, and infectious diseases for fewer than 3 weeks.

#### Statistical Analysis

Shapiro–Wilk test will be used to determine normality. For parametric distributions, dependent or independent *t* tests will be performed. To determine differences between categorical variables, the chi-squared test will be used. For nonparametric distributions, we will use the Wilcoxon test and Mann–Whitney *U* test to compare nonrelated and related means, respectively. A generalized estimating equations (GEE) model will be used to investigate multivariate repeated measurements. The Hayes method will be used to analyze the mediators (52), and the multivariate regression will be used to analyze the confounders. *Post hoc* analyses will be performed using the Bonferroni test.

The data will be expressed as mean ± standard deviation (SD), and a *p* value less than or equal to 0.05 will be considered to be statistically significant (two-tailed). The statistical analyses will be performed using the SPSS version 20.0 software package (IBM^®^, New York, USA).

### Procedure

The study will be disseminated through the presentation of its protocol in meetings with the outpatient services of the HCPA that refer patients to the psychiatry service, such as the internal medicine, pain, and bariatric surgery outpatient clinic.

**Figure 1:**
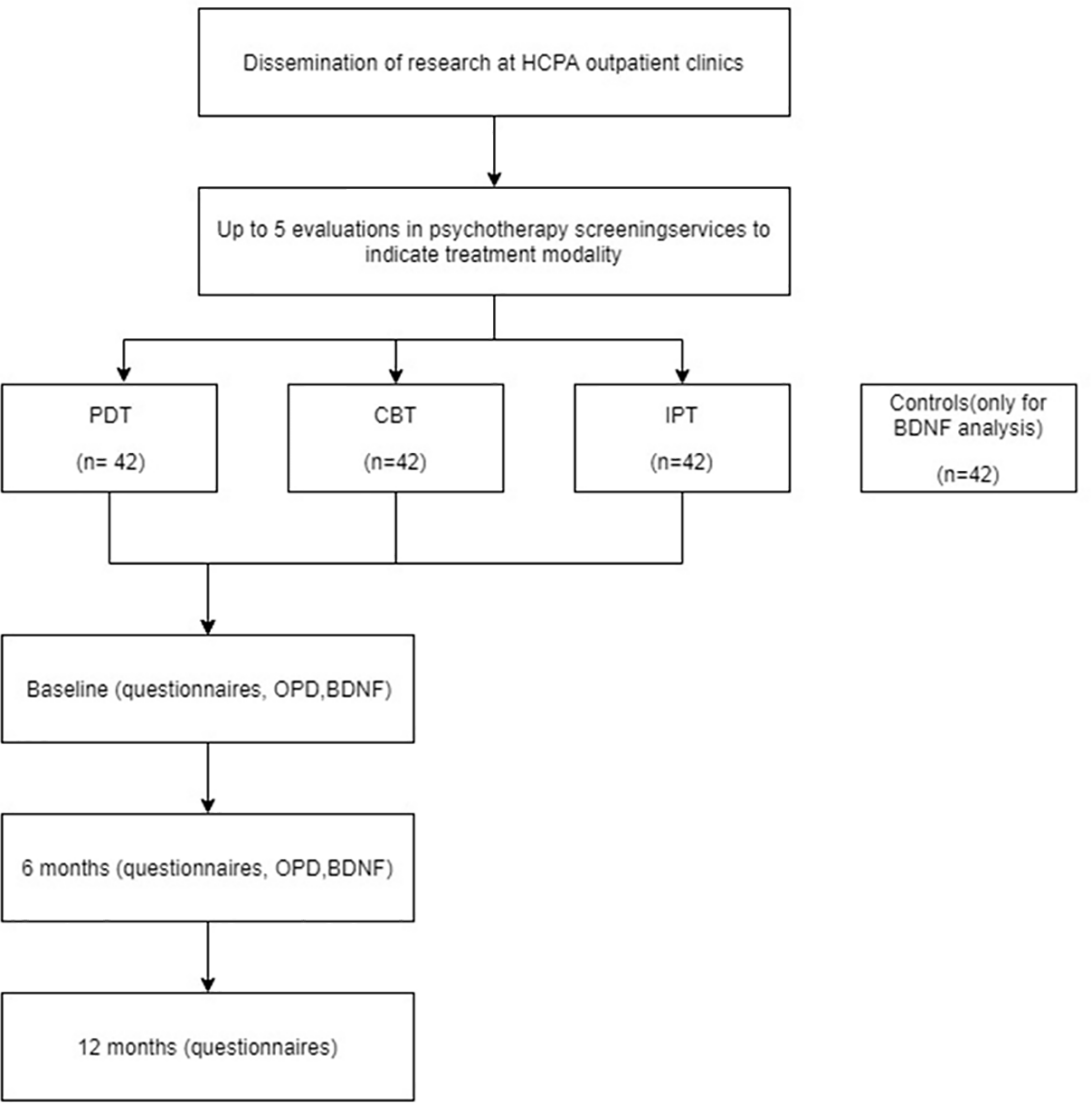
Study design.

The researchers will not participate in the indication of treatment of patients or in their assistance. Patients will be sought weekly in the HCPA outpatient medical records and through contact with resident physicians screening new patients. Those who fall into the eligibility criteria will be contacted by phone by the research assistant and invited to participate. It will be explained that refusal to participate will not entail any loss to the treatment. If they agree to participate, they will be asked to attend the HCPA Clinical Research Center (CPC-HCPA), where they will receive the informed consent form, sociodemographic interview, OPD interview, BDNF blood collection, and protocol with self-administered questionnaires. The consent form and OPD will be applied by the researchers. Patients will be contacted by telephone for the follow-up of 6 and 12 months (Figure 1).

#### Location

This study will be conducted in the city of Porto Alegre in the state of Rio Grande do Sul in far-southern Brazil. This region has Portuguese, Italian, German, and African influence. It has a population of 1.4 million, and it is the 10th most populous city in Brazil. Its ethnic distribution is as follows: White (79.2%), Black (10.2%), and Pardo (multiracial) people (10.6%). Its religious distribution is as follows: Catholic (83%), Protestant (9%), and others and atheists (8%). Brazil is a country with a high level of religiosity, and it is common for people to associate improvements in their health as being due to their faith (53). The role of religiosity in psychotherapies has not yet been clarified. According to the World Crime Index, Brazil ranks 9th, with Porto Alegre and São Paulo in the 11th and 14th place, respectively; so many patients experience a high level of urban violence and, consequently, severe life stressors related to this.

## Discussion

The only naturalistic longitudinal study that we found concluded that there was no significant difference in outcomes between therapies, as well as among therapists. However, these results need to be viewed with caution given some limitations related to loss, incomplete data, and the lack of long-term follow-up. Our study used direct contact with patients, which increased the number of variables and collection of BDNF as a possible biomarker in response to psychotherapy intervention.

Historically, studies in psychotherapy have been difficult to perform because they involve multiple factors and variables that are interrelated. Many variables may act as only associated factors, and others (e.g., mediators, predictors and even as confounders) may have varying effect sizes. We had difficulty defining some variables as mediators or confounders because the mechanism by which psychotherapies work is a matter of debate (54). We are interested in determining what is common to all psychotherapies. Therefore, we relied on published studies (16) and clinical experience.

Regarding the biological factors, studies of biomarkers such as BDNF may provide insights into the possible mechanisms of illness and recovery from serious disorders such as depression, bipolar disorder, and schizophrenia (22). A few studies have evaluated this neutrotrophin in the context of psychotherapies (29, 55). However, the mechanisms by which long-term psychotherapies produce changes in neuronal circuits and biomarkers, such as BDNF, have not yet been determined. In addition, most existing studies are characterized by follow-up durations of fewer than 12 weeks. As a result, they may be unable to detect changes that occur more slowly than those observed in BDNF with psychotropic drugs (56).

Considering the severity profile of patients referred to a tertiary medical center and the level of experience of resident physicians, the magnitude of improvement is lower than in other more favorable clinical settings. This improvement should be more pronounced in the 12-month follow-up considering the long-term effects of psychotherapies. The level of therapeutic alliance may possibly mediate this improvement. We believe that it will also reflect patients’ psychosocial factors and increase BDNF.

Limitations of this study include the heterogeneity of the training and experience of the resident psychiatrists and the absence of evaluation from their point of view in the psychotherapeutic process. As a study is naturalistic, there is no way to control the impact of the researchers’ interviews. We will collect a series of demographic data, use of medications, and number of sessions to control possible confounders, but we know that because it is an open study, there is no way to control all differences between groups. Also, psychotherapy studies often have large amounts of losses (16). To control this bias, we will compare the clinical and demographic characteristics of the lost sample with those that continue in relation to the baseline. As for the multiplicity of diagnoses that may be found, to homogenize the sample, we will consider for inclusion the BDI value >15.

Studies such as this one seek to contribute to the foundations of public health policies and investments for the treatment of patients with severe mental illnesses who are at risk of hospitalization and suicide. These types of investigations also seek to promote mental health, rehabilitation, and social reintegration. Understanding the mediating and biological mechanisms of the psychotherapeutic process can help to optimize these interventions and facilitate their indication according to the psychopathological profile and the severity of the patients.

## Ethics Statement

This project was approved by the Ethics Committee of Hospital de Clínicas de Porto Alegre (GPPG–HCPA no 97-2015), and it is recognized by the CONEP Conselho Nacional de Ética em Pesquisa—National Council of Ethics in Research.

## Author Contributions

LG and NR codeveloped the project. NR coordinated the project in HCPA. LG, GB, and CR were responsible for implementing, conducting, and collecting project data.

## Funding

This study was financed in part by the Coordenação de Aperfeiçoamento de Pessoal de Nível Superior–Brasil (CAPES)—Finance Code 001 and by Hospital de Clínicas de Porto Alegre Research Incentive Fund (FIPE).

## Conflict of Interest Statement

The authors declare that the research was conducted in the absence of any commercial or financial relationships that could be construed as a potential conflict of interest.
